# Cloning, recombinant expression, and characterization of a *Rhipicephalus microplus* carboxylesterase.

**DOI:** 10.12688/gatesopenres.16245.1

**Published:** 2024-08-19

**Authors:** Michel Labuschagne

**Affiliations:** 1 Research Innovation, Clinglobal, Tamarin, 90903, Mauritius; 2 Research Innovation, Clinomics, Bloemfontein, Free State, 9338, South Africa; 3 Department of Microbiology and Biochemistry, University of the Free State Faculty of Natural and Agricultural Sciences, Bloemfontein, Free State, 9300, South Africa

**Keywords:** Rhipicephalus microplus; Carboxylesterase; Recombinant expression; Purification; Resistance; Pichia pastoris

## Abstract

**Background:**

The *Rhipicephalus microplus* carboxylesterase (CBE) is involved in synthetic pyrethroid (SP) hydrolysis and historic evidence suggests that a non-synonymous mutation (Asp374Asn) in CBE is associated with increased resistance towards SP-based acaricides. Functional expression and characterization of the wild-type and mutant CBE is required to understand the impact of the mutation on SP-based resistance.

**Methods:**

The *R. microplus* CBE gene was cloned and functionally expressed in *Pichia pastoris* following the removal of the native signal peptide. Site directed mutagenesis was used to introduce the Asp374Asn substitution.

**Results:**

Functional expression, characterization, and purification of both wild-type and mutant *R. microplus* CBE proteins was achieved using affinity chromatography under native conditions.

**Conclusions:**

This report provides the necessary information for the tick research community to produce recombinant tick derived CBE proteins and to characterize the recombinant proteins towards substrates of interest.

## Introduction

The Asian blue tick *Rhipicephalus microplus* is an economically important tick responsible for significant economic losses in the cattle industry ^1^. Chemical control strategies have been used effectively since the 1950s to manage tick populations. However, *R. microplus* populations have developed resistance to all the classes of commercial acaricides currently in use. Resistance mechanisms to acaricides can be classified into affinity differences for target binding, enhanced acaricide degradation, and reduced acaricide acquisition from the environment ^2^. Synthetic pyrethroids (SPs) are one of the major classes of acaricides, accounting for approximately 17% of the total insecticide market in 2007, with annual sales exceeding USD 1.5 billion ^3^. SP resistance mechanisms have been identified, with sodium channel *kdr* mutations being the most prevalent in SP-resistant phenotypes ^4, 5^. Another resistance mechanism in which SP is enzymatically hydrolyzed by *R. microplus* carboxylesterase (RmCBE) was reported by Jamroz and coworkers ^6^, and the occurrence of an Asp374Asn-encoded mutation in RmCBE was statistically linked to SP resistance (Guerrero *et al*., 2002). Differential gene expression between isolates was ruled out as a factor contributing to resistance, but attempts to produce enough active recombinant RmCBE in *Escherichia coli* and *Pichia pastoris* for the characterization of both the wild-type and mutant versions were unsuccessful ^7^. Translational analysis of acaricide resistance targets from LPT isolates indicated the presence of the Asp374Asn mutation in 14 out of 16 synthetic pyrethroid-resistant isolates from East and West Africa (manuscript in preparation). The recombinant expression, purification, and characterization of the wild-type and mutant versions of this carboxylesterase will provide evidence on whether the mutant version results in increased activity toward a range of synthetic pyrethroids and organophosphates.

The heterologous expression of recombinant proteins enables controlled production under standard parameters and allows for the addition of sequences for standardized downstream purification of recombinant proteins. *Pichia pastoris* is an attractive recombinant heterologous host due to its ability to secrete recombinant proteins into the extracellular medium ^8^. Although it has been used to produce active RmCBE, this process has resulted in low yields even after significant optimization ^7^.

We attempted to express the complete open reading frame (ORF) encoding RmCBE using *P. pastoris* with different signal peptides (α-factor and putative native RmCBE signal peptide) but were unable to produce detectable recombinant protein by SDS‒PAGE. Finally, we removed the putative native signal peptide of RmCBE, fused the putative mature protein with the α-factor signal peptide, and drove recombinant expression using the methanol-inducible *AOX1* promoter in *P. pastoris*.

## Methods

### Cloning and sequencing of RmCBE

All oligonucleotides used in this study were obtained from either IDT or Inqaba Biotechnical Industries. Restriction and DNA modification enzymes were obtained from either New England Biolabs or Thermo Fisher Scientific. Standard molecular biological techniques were performed according to the manufacturer’s recommendations. Total genomic DNA (isolated from the Clinvet South Africa-susceptible *R. microplus* isolate) served as a template for 3 sets of PCR primers to amplify the full ORF of the RmCBE. The primer sets Rm_CBE_E1-1F(EcoRI) + Rm_CBE_E1-1R; Rm_CBE_E2-1F + Rm_CBE_E2-1R; and Rm_CBE_E3-1F + Rm_CBE_E3-1R (XbaI) ( Table 1) were used to amplify the 3 exons encoding the full ORF using Thermo Scientific™ Phusion Plus PCR Master Mix according to the manufacturer’s instructions (Thermo Fisher Scientific; Catalogue number: F631S). The PCR products were analyzed using agarose gel electrophoresis, and the expected PCR products were excised from the gel and purified using the GeneJET Gel Extraction and DNA Cleanup Micro Kit (Thermo Fisher Scientific; Catalogue number: K0831). The purified PCR products were phosphorylated, followed by ligation of E1 and E2. This product served as template during the next round of PCR using the primers Rm_CBE_E1-1F (EcoRI) and Rm_CBE_E2-1R to amplify the fusion between E1 and E2. The fused product was gel-purified, phosphorylated, and ligated to phosphorylated E3, followed by PCR amplification using the primers Rm_CBE_E1-1F (EcoRI) and Rm_CBE_E3-1R (XbaI) to generate PCR fusion products encoding the complete RmCBE ORF flanked by the *Eco*RI and *Xba*I restriction sites for expression cloning. The E1+E2+E3 PCR product was phosphorylated and ligated into the pSMART-HC Kan cloning vector (Lucigen) and used to transform chemically competent *E. coli*. Plasmid DNA from 2 independent clones was subjected to Sanger sequencing on both DNA strands, confirming a perfect match to the CVSA RmCBE reference sequence ( *de novo* genome assembly manuscript under review) and designated pSMART-HC_Kan+RmCBE. The CVSA RmCBE was submitted to GenBank under accession OR124739.

**Table 1.** Oligonucleotides used in this study.

Primer name	Sequence (5'-3')
Rm_CBE_E1-1F(EcoRI)	GAATTCATGGCGGTGAAAGCAGCTGTGCTG
Rm_CBE_E1-1R	CTGTGGGCATGCAGTTCGCGTGGA
Rm_CBE_E2-1F	ATAGAGATGCAACTGGTCATCATGAAC
Rm_CBE_E2-1R	CCATTTTCGCTGAAGCTTCCCAGCATC
Rm_CBE_E3-1F	GCGTCCCGAGCTGCCCAGCAAGCAGAAG
Rm_CBE_E3-1R(XbaI)	TCTAGAGCGAAGAGTGACTTCCAGCGCTCGCATTG
Rm_CBE-2F(EcoRI)	GAATTCGTCATGGCGGTGAAAGCAGCTGTGCTG
RmCBE_mut-1F	AATTCTCTTCGCGCGGCTCTATCATCATG
RmCBE_mut-1R	CTTGAGTTTCTCTGGATTGCTTCCGTC

The complete ORF encoding RmCBE was analyzed using signalP_6.0 ( https://services.healthtech.dtu.dk/services/SignalP-6.0/). Subsequently, the primer pair Rm_CBE-2F (EcoRI) and Rm_CBE_E3-1R (XbaI) was used to amplify the mature RmCBE (excluding the predicted signal peptide) encoding sequence from the pSMART-HC Kan vector, which contains the verified full-length ORF encoding sequence. The resulting PCR product was phosphorylated, cloned, and inserted into pSMART-HC Kan. After verification by Sanger sequencing, the construct was designated pSMART-HC_Kan+RmCBE-sigP.

Site-directed mutagenesis was performed using the phosphorylated primer pair RmCBE_mut-1F and RmCBE_mut-1R to introduce the Asp374Asn mutation in the resulting PCR product using pSMART-HC_Kan+RmCBE-sigP as a template. The PCR products were subjected to *Dpn*I treatment, followed by self-ligation and transformation of competent *E. coli*. Plasmid DNA isolated from transformants was analyzed by *Eco*RI restriction digestion to confirm the presence of the mutation. Additionally, Sanger sequencing was performed to ensure that the ORF matched the wild-type sequence, except for the site-directed mutation.

### Expression and purification of recombinant RmCBE (rRmCBE)

DNA encoding the wild-type RmCBE complete and mature ORF sequences was subcloned using *Eco*RI and *Xba*I to release the RmCBE-encoding fragments. These fragments were then ligated into the pPIC-B and pPICZα-A expression vectors to ensure in-frame fusion with the c-myc epitope and a hexa-histidine tag at the 3’-end (Invitrogen™ EasySelect™ *Pichia* Expression Kit; Catalogue number: K174001). DNA encoding the Arg374Asn-mutated RmCBE mature ORF sequence was digested with *Eco*RI and *Xba*I, resulting in the release of two fragments: a 519 bp fragment flanked by *Eco*RI and *Xba*I on the 5’ and 3’ ends, respectively, and a 1065 bp fragment flanked by *Eco*RI on both ends. The 519 bp fragment was ligated into *Eco*RI- and *Xba*I-linearized pPICZα-A, and the resulting plasmid was digested with *Eco*RI, dephosphorylated and used as an acceptor vector for the 1065 bp fragment. Ligation mixtures were used to transform competent *E. coli* for subsequent plasmid DNA preparation. Restriction analysis was performed to verify cloning, and the expression construct was subjected to Sanger sequence analysis. Approximately 1 µg of each correct expression construct (pPICZα-A+RmCBE/pPICZ-B+RmCBE/pPICZB+RmCBE-sigP/pPICZα-A+RmCBE-sigP/pPICZα-A+RmCBE-sigP_MUT) was linearized using *Sac*I prior to transformation. Approximately 500 ng of the resulting linear expression cassettes were then used to transform electrocompetent *P. pastoris* wild-type cells (X33) by electroporation following a condensed protocol ^9^. Transformed cells were subsequently plated onto YPD media supplemented with zeocin (100 µg/ml final concentration).

The procedures described in the EasySelect™ Pichia Expression Kit manual were used to assess the transformants. The transformants were streaked onto fresh YPD plates supplemented with zeocin and subjected to PCR screening to confirm the integration of the expression cassette at the intended locus. Transformants with correctly integrated expression cassettes were subjected to small-scale expression studies using 25 ml of BMGY medium for biomass generation, followed by induction in 25 ml of BMMY medium for 96 hours. Absolute methanol was added every 24 hours to a final concentration of 0.5% (v/v). After the cultures were centrifuged (3000 *rcf* for 5 min at 25°C), the cells and supernatants were separated and frozen at -80°C.

The supernatants (representing 13 µl of crude supernatant) were analyzed on Invitrogen™ Bolt™ Bis-Tris Plus Mini Protein Gels, 4–12%, 1.0 mm, WedgeWell™ format (Thermo Fisher Scientific; Catalogue number: NW04125BOX) using Invitrogen™ Bolt™ MES SDS Running Buffer (Thermo Fisher Scientific; Catalogue number: B0002) and were Coomassie-stained with 25 ml SimplyBlue ^TM^ Safestain (Thermo Fisher Scientific; Catalogue number: LC6065). The cells were treated under mildly alkaline conditions ^10^, and 3 µl of cell lysate was loaded for each transformant and analyzed via SDS‒PAGE as indicated above.

Culture supernatants (7.5 ml) were subjected to immobilized metal affinity chromatography (IMAC) using 1 ml Thermo Scientific™ HisPur™ Ni-NTA Resin slurry and the batchwise method as per the manufacturer’s recommendations under native conditions (Thermo Fisher Scientific; Catalogue number: 88221). The different fractions were analyzed via SDS‒PAGE, and the protein concentrations were determined using the Pierce™ BCA Protein Assay Kit (Thermo Fisher Scientific; Catalogue number: 23227).

Known volumes (10 µl) of purified RmCBE protein, culture supernatants, and BSA standard dilutions were subjected to SDS‒PAGE analysis as indicated above. The gel images were captured with a Samsung S23 smartphone camera and analyzed with ImageJ ( https://imagej.net/ij/) according to the one-dimensional electrophoretic gel analysis workflow. The areas under the peaks were quantified using the BCA-quantified purified rRmCBE WT and MUT proteins. These quantities were then used to calculate the volumetric productivity of the RmCBE-expressing *P. pastoris* transformants.

### Characterization of recombinant RmCBE

Crude supernatants were diluted 10-, 100-, and 1000-fold with 50 mM Tris-HCl (pH 7.4). Recombinant protein activity assays were performed using *p*-nitrophenyl acetate (Sigma-Aldrich; Catalogue number: N8130) as a substrate, according to basic protocol 1 ^11^. The 100-fold dilution exhibited linearity over the 5-minute monitoring of the product. Supernatants from both RmCBE-expressing and control strains (transformed with an empty plasmid) were subjected to 100-fold dilution, with a buffer-only control included to account for chemical hydrolysis. The reactions were performed in a final volume of 500 µl at 37°C for 5 min, and A _405_ readings were recorded every 7 seconds to monitor the formation of the *p*-nitrophenol product using a Jenway 7205 spectrophotometer. All hydrolytic reactions were performed in triplicate. One-way ANOVA was performed using the triplicate endpoint absorbance readings obtained during the assay, followed by a post hoc Tukey HSD test.

The complete α-factor:RmCBE-sigP_WT/MUT:c-myc_6xHis peptide sequence was submitted to SWISS-MODEL ( https://swissmodel.expasy.org/), and the best fit template (AlphaFold DB Q9U6M8_RHIMP representing the *R. microplus* CBE; 97.71 protein identity) was selected. The resulting model was visualized and annotated in Yasara View ^12^ to indicate the Asp374Asn position and the catalytic triad members of the active site. Both the WT and MUT models were superimposed to check for structural variation.

## Results and discussion

### Cloning and sequencing of RmCBE

The amplicon-fused ORF DNA sequence encoding the CVSA RmCBE was identical to the RmCBE sequence obtained from the genome sequence (manuscript currently under review) and showed ≥97.7% protein identity to the *R. microp*lus CBE-deduced peptide sequences in GenBank ( Table 2).

**Table 2.** Deduced amino acid identity between the CVSA RmCBE and RmCBE sequences available in GenBank.

	AAF00497	XP_037272683	AOA32871	ALD51318	ALD51320	ALD51319	CVSA RmCBE
AAF00497		99.8	96.6	96.6	96.4	95.8	97.9
XP_037272683	99.8		96.4	96.4	96.2	95.6	97.7
AOA32871	96.6	96.4		99.6	99.4	98.9	98.7
ALD51318	96.6	96.4	99.6		99.4	98.9	98.7
ALD51320	96.4	96.2	99.4	99.4		99	98.5
ALD51319	95.8	95.6	98.9	98.9	99		97.9
CVSA RmCBE	97.9	97.7	98.7	98.7	98.5	97.9	

### Expression and purification of rRmCBE

**Figure 1.**
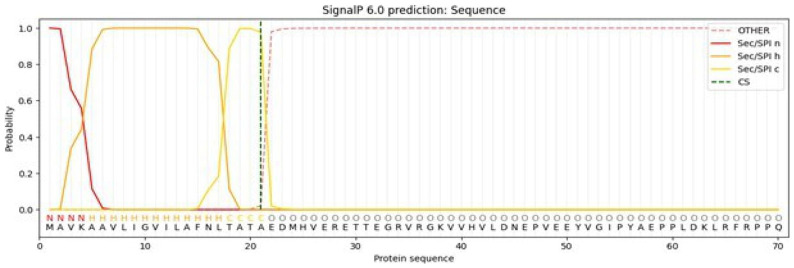
SignalP_6.0 signal peptide prediction for the deduced protein sequence encoded by the CVSA RmCBE ORF.

Signal peptide identification using SignalP_6.0 revealed a putative signal peptide encoded by residues 1 to 21 ( Figure 1). Primers were designed to utilize the native signal peptide (pPICZ-B) or remove the native signal peptide and fuse residue 21 to the α-factor signal peptide in the pPICZα-A vector.

**Figure 2.**
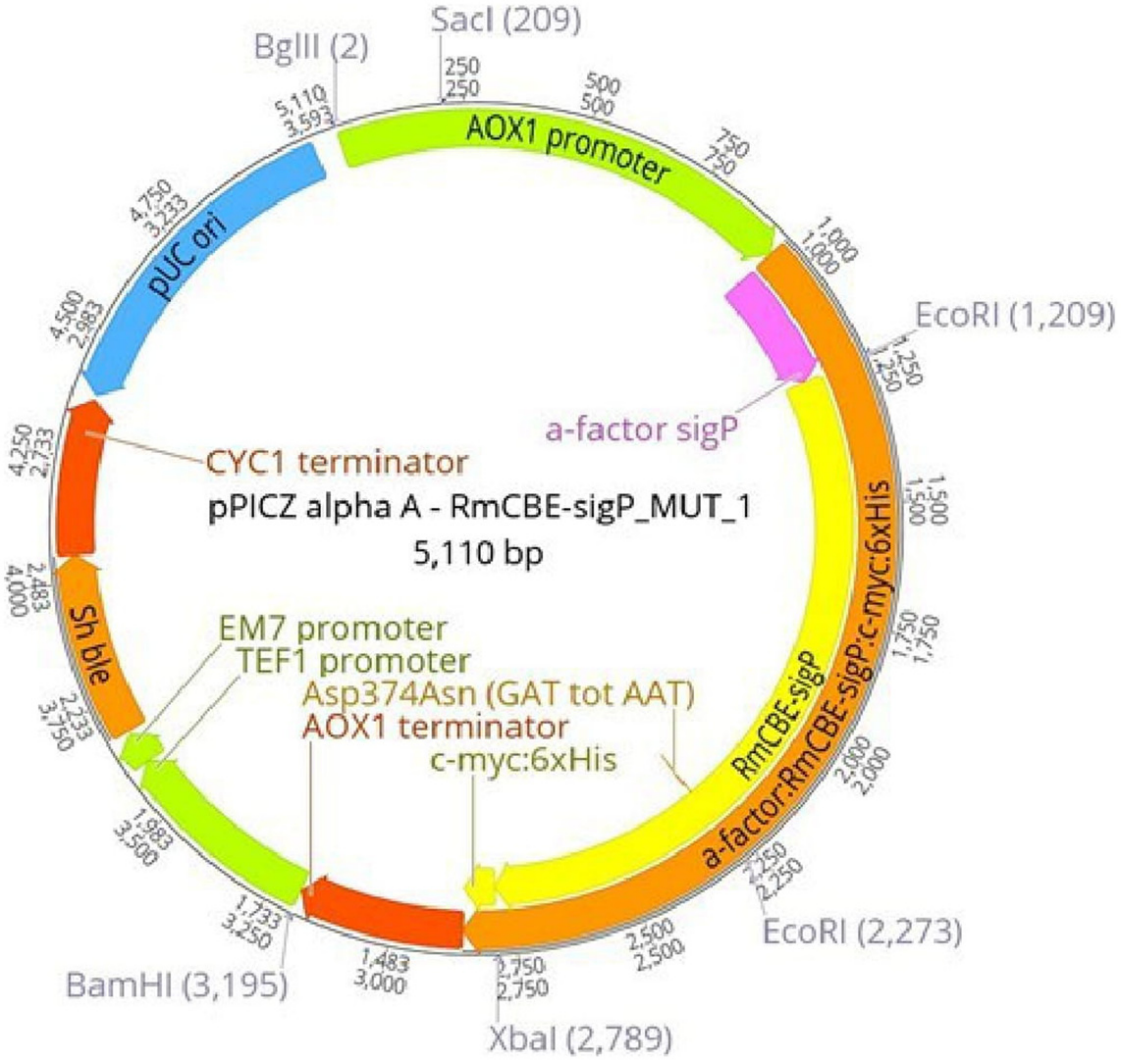
pPICZα-A vector containing the CVSA RmCBE-sigP_MUT gene under the control of the *AOX1* promoter.

All CVSA RmCBE-encoding genes and derivatives were cloned and inserted into pPICZ vectors for methanol-induced expression in *P. pastoris* ( Figure 2). The *Sac*I linearized vectors were used to transform wild-type *P. pastoris* (X33) via genomic integration at the *AOX1* locus, which was confirmed by PCR (data not shown).

Multiple transformants obtained from each construct (6 randomly selected) were subjected to small-scale protein expression studies with biomass cultivation in phosphate-buffered BMGY medium for 24 hours followed by induction with 0.5% (v/v) methanol every 24 hours for 96 hours in phosphate-buffered BMMY medium. Supernatant and crude cell lysates were evaluated using Coomassie-stained SDS‒PAGE to detect the presence of the expected rRmCBE product. No distinct extracellularly expressed protein bands of the expected size were observed for the pPICZ-B+RmCBE or pPICZα-A+RmCBE transformants subjected to small-scale protein expression with methanol induction for 96 hours after biomass generation (data not shown). The intracellular accumulation of proteins was also assessed, and no difference in the SDS‒PAGE protein profiles was detected compared to those of the empty plasmid control transformant (data not shown).

**Figure 3.**
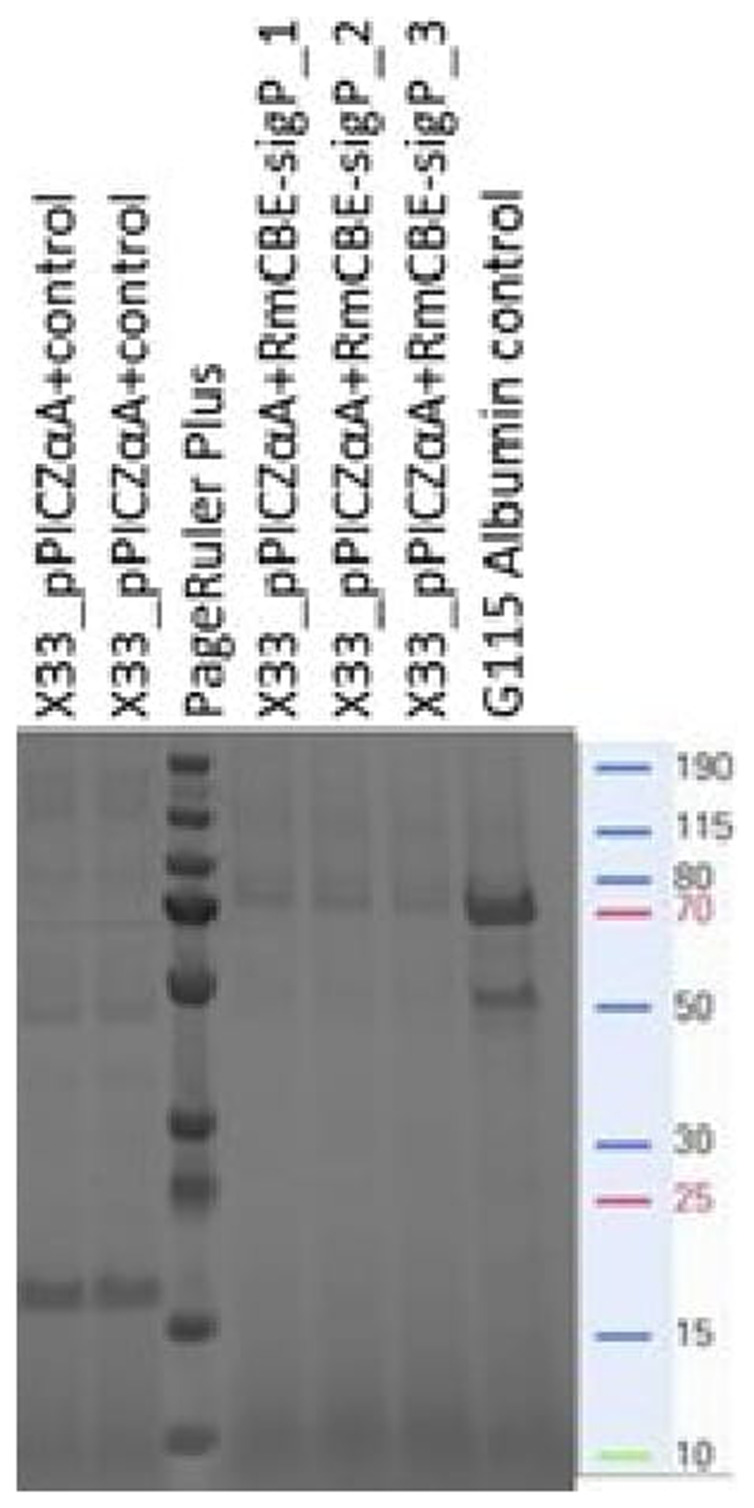
Coomassie-stained SDS‒PAGE of supernatants obtained from transformants with an expression cassette for an in-house protein (lanes 1 and 2), α-factor:RmCBE devoid of the native signal peptide fusion (lanes 4–6) and the GS115 control isolate (secreting a 67 kDa recombinant albumin) supplied with the Pichia expression kit (lane 7). The PageRuler Plus approximate sizes are also indicated.

Analysis of the supernatant from transformants devoid of the native signal peptide and fused in frame with the α-factor signal peptide revealed the presence of an approximately 70 kDa protein band in the supernatants of transformants harboring the pPICZα-A+RmCBE-sigP vector cassette integrated into the genome ( Figure 3). The theoretical expected size of the recombinant protein containing the α-factor signal peptide fused to RmCBE and the c-myc_6xHis tag was 70.9 kDa, whereas the recombinant protein with the processed α-factor signal peptide should have a theoretical molecular weight of 61.4 kDa.

**Figure 4.**
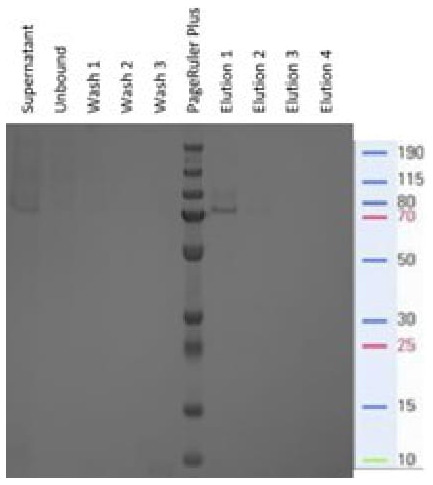
Coomassie-stained SDS‒PAGE of IMAC fractions obtained during the purification of the supernatant from the RmCBE-sigP-producing transformants.

The resulting supernatant exhibiting the approximately 70 kDa protein band was subjected to immobilized metal affinity chromatography (IMAC) using Ni-NTA under native conditions via a batchwise approach. SDS‒PAGE analysis of the different fractions collected during IMAC revealed efficient binding of the approximately 70 kDa protein from the supernatant (comparing the supernatant to the unbound fraction) and elution of a distinct band of approximately 70 kDa, with most of the protein eluting during the first elution fraction ( Figure 4).

IMAC purification under native conditions confirmed that the 6xHis tag was accessible to interact with the Ni-NTA resin, and the observed size revealed that the recombinant RmCBE (rRmCBE) still contained an α-factor secretion signal (approx. 9.5 kDa) fused to the RmCBE-sigP-encoding peptide and c-myc_6xHis tag (61.4 kDa).

### Characterization of recombinant RmCBE

**Figure 5.**
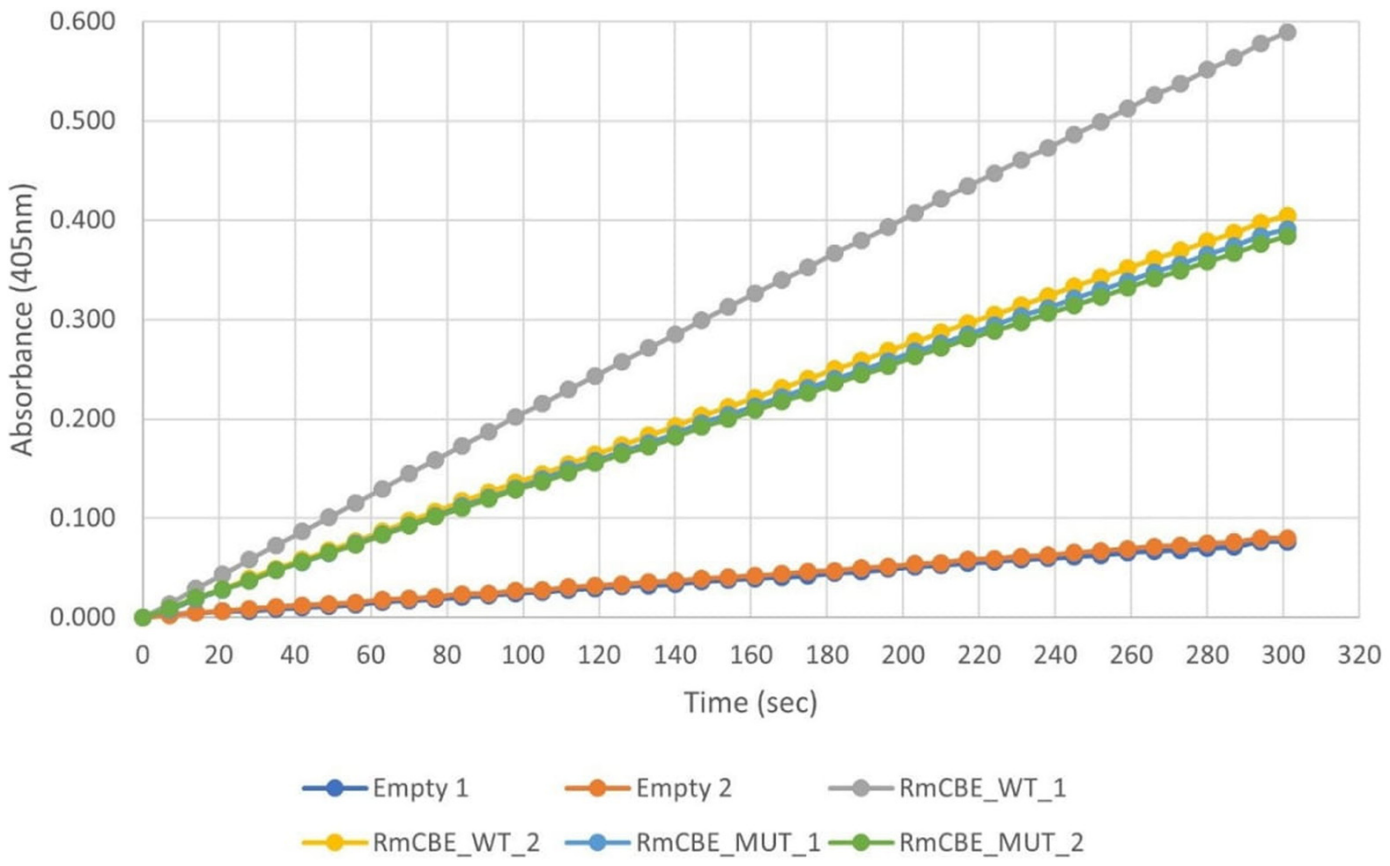
Spectrophotometric detection of *p*-nitrophenolate over time using a 100-fold dilution of culture supernatants. Buffer-only controls were used as reaction blanks prior to data generation to account for chemical hydrolysis.

Supernatants (diluted 100-fold) from two isolates secreting the α-factor RmCBE-sigP_WT/MUT:c-myc-6xHis protein, along with two control isolates containing empty plasmids, were subjected to CBE activity assessment using *p*-nitrophenyl acetate as a substrate. Hydrolytic product formation of *p*-nitrophenolate was monitored spectrophotometrically at 405 nm every 7 seconds for 5 minutes ( Figure 5).

Compared with those of the empty vector controls, the culture supernatants derived from the RmCBE-harboring transformants showed at least a 4.8-fold increase in *p*-nitrophenolate formation. The RmCBE_WT_1 transformant exhibited a >1.5-fold increase in activity compared to the RmCBE_WT_2, RmCBE_MUT_1, and MUT_2 transformants, most likely due to increased cassette copy number integration into the genome. End-point analysis indicated that all the rRMCBE-expressing transformants produced significantly more product (p<0.01) than the transformants containing empty expression cassettes. Specifically, the RmCBE_WT_1 transformant produced significantly more product (p<0.01) than the other RmCBE transformants. There was no significant difference in end-point product formation detected between the supernatants derived from the RmCBE_WT_2, RmCBE_MUT_1, and RmCBE_MUT_2 transformants.

**Figure 6.**
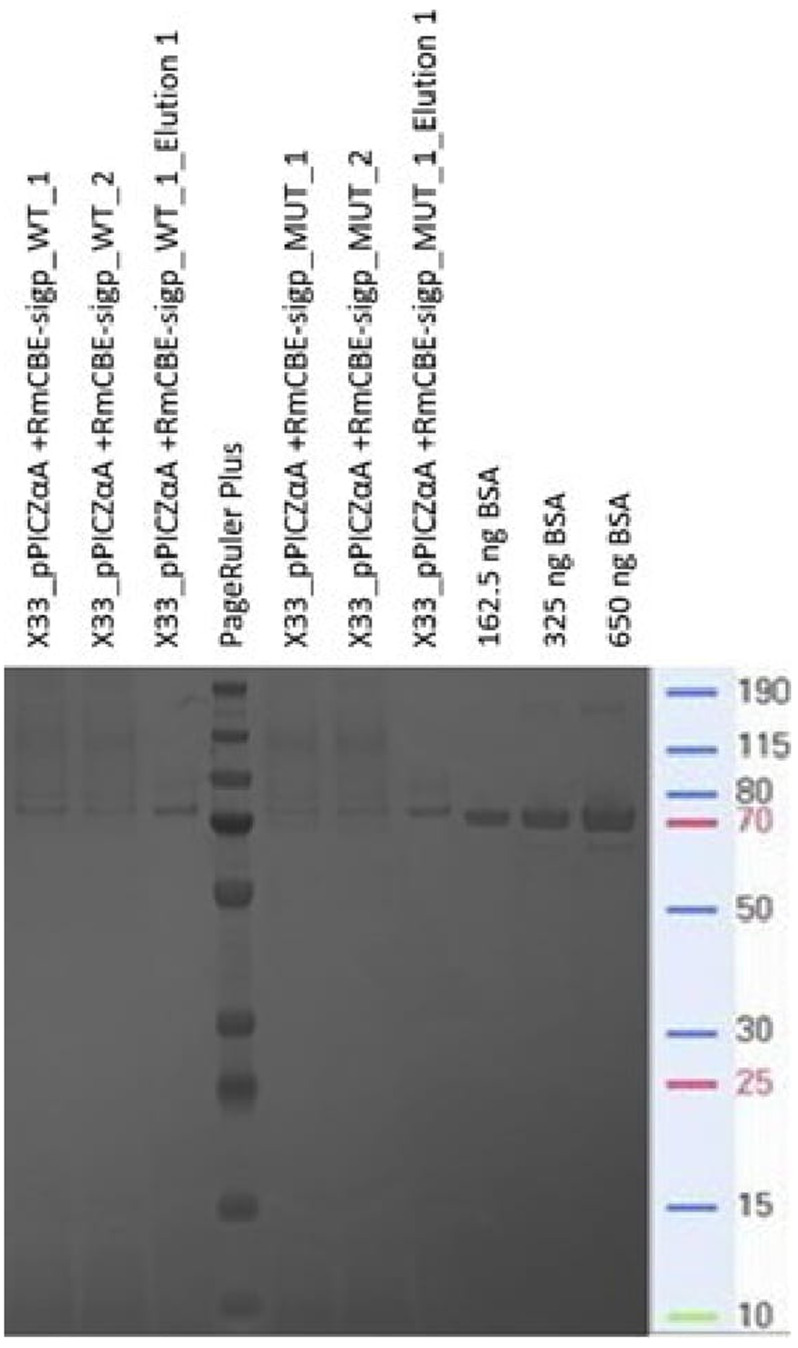
A Coomassie-stained SDS‒PAGE gel showing 6.5 µl of crude supernatant from the WT (lanes 1 and 2) and MUT (lanes 5 and 6) transformants, with unknown quantities, alongside WT (lane 3) and MUT (lane 7) purified proteins of known quantities. BSA, which was loaded in known quantities, was included as a control signal for image analysis and quantification purposes.

The specific activity of the WT and MUT proteins purified by IMAC could not be determined using the *p*-nitrophenyl acetate substrate due to the presence of high levels of imidazole (250 mM) in the elution buffer, which causes chemical hydrolysis of the substrate to *p*-nitrophenolate (Bender and Turnquest, 1956). However, the IMAC-purified samples were quantified using the BCA assay. Equal volumes of purified CVSA RmCBE WT and MUT proteins (10 µl each) were loaded alongside 10 µl of BSA protein (162.5, 325, and 650 ng per lane), as well as 10 µl of crude supernatant analyzed in the hydrolysis reaction ( Figure 5). The resulting gel image was quantified using ImageJ, and the signals of the purified RmCBE were used to convert the 70 kDa bands in the supernatant signals to protein quantities ( Figure 6). Known BSA controls were used as internal controls to assess the accuracy of the conversion factor. The conversion of 162.5 ng of BSA signal to protein was measured at 100.04% of the theoretical quantity loaded ( Table 3).

**Table 3.** ImageJ quantification and resulting calculations based on quantified data.

Sample	ImageJ protein quantity (ng)	BCA measured protein quantity (ng)	ImageJ quantity:BCA quantity	Specific activity (µmol product formed/min/mg protein)	Shake flask rRmCBE production (mg/L)
rRmCBE_WT_1	34.60	ND	NA	8.303	5.32
rRmCBE_WT_2	24.21	ND	NA	7.679	3.72
rRmCBE_WT_1 Elution	62.86	59.54	1.06	NA	NA
rRmCBE_MUT_1	17.18	ND	NA	9.837	2.64
rRmCBE_MUT_2	20.26	ND	NA	8.340	3.12
rRmCBE_MUT_1 Elution	65.27	69.14	0.94	NA	NA
162.5 ng BSA	162.57	162.5	1.00	NA	NA

**Figure 7.**
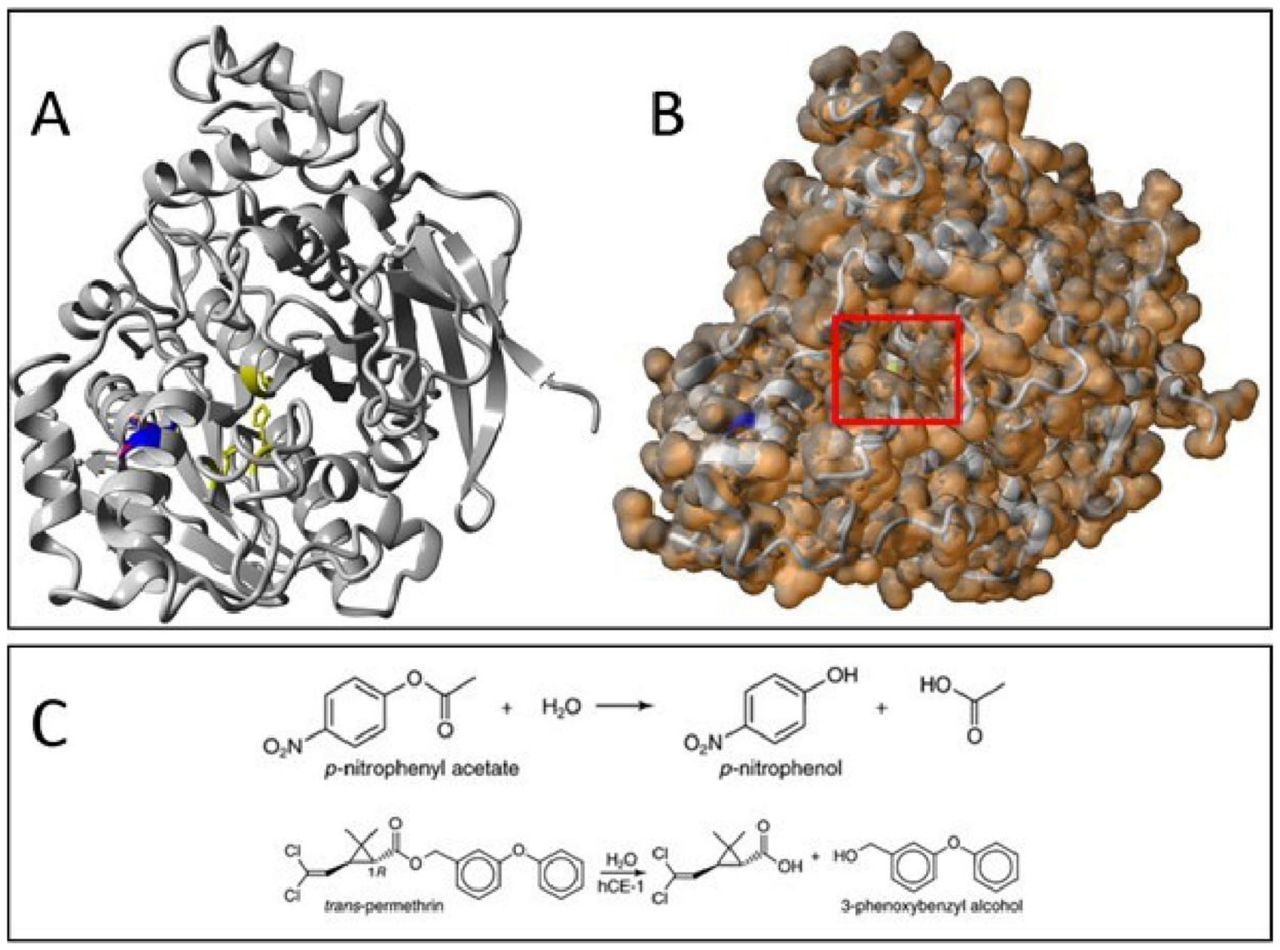
Superimposed 3D protein models of the CVSA RmCBE WT and MUT proteins with the Asp374Asn mutation (Asp, red; Asn, blue) and the active site catalytic triad members (yellow) displayed as balls and sticks ( **A**). The solvent-accessible surface overlay indicating the tunnel toward the active site (red box; **B**) CBE substrate and hydrolytic products used in this study ( *p*-nitrophenyl acetate) and a typical insecticidal pyrethroid ( *trans*-permethrin) ( **C**; taken from Ross and Borazjani, 2007).

ImageJ quantification of the 70 kDa protein bands ( Table 3) in the supernatants indicated that the amount of RmCBE_WT_1 was 1.43-fold greater than that of RmCBE_WT_2, which is in concordance with the increased hydrolytic activity observed ( Figure 5). Specific activity determinations toward the universal CBE substrate *p*-nitrophenyl acetate, based on the ImageJ protein quantities and the hydrolytic activities, indicated no apparent difference between the WT and MUT versions of the rRmCBE proteins assessed. The presence of the mutation ( Figure 7A; blue residue) in the cap structure, which forms part of the solvent accessible tunnel toward the active site (catalytic triad residues in yellow) ( Figure 7 A and B), might alter substrate binding pockets specific to SP-derived substrates.

The smaller chemical structure of *p*-nitrophenyl acetate (relative to SP derivatives; Figure 7C) is unlikely to be affected by the Asp374Asn mutation, which is located close to the conserved putative substrate binding pockets on a relatively distant α-helix. The effect of the mutation will only become evident with the assessment of SP derivatives currently in commercial use.

The specific activity of the supernatant rRmCBE toward *p*-nitrophenyl acetate ( Table 3) is comparable to that of commercially available His-tagged recombinant human carboxylesterase 1 derived from mammalian cell culture (6 µmol/min/mg; https://www.rndsystems.com/products/recombinant-human-carboxylesterase-1-ces1-protein-cf_4920-ce).

Quantification of the rRmCBE protein in the supernatant allowed the calculation of the volumetric productivity of the secreted rRmCBE by the recombinant host cultures evaluated, which ranged between 2.64 and 5.32 mg/L under unoptimized shake flask culture conditions ( Table 3).

## Conclusion

The CVSA RmCBE WT and Asp374Asn mutant-encoding genes were successfully expressed and secreted extracellularly from *P. pastoris*. The recombinant proteins were approximately 70 kDa in size, suggesting that the heterologous α-factor secretion signal was not processed and formed part of the secreted recombinant protein. Activity determination using *p*-nitrophenyl acetate revealed that the culture supernatants of the transformants harboring the RmCBE WT and MUT expression cassettes exhibited activity toward the substrate and resulted in *p*-nitrophenolate product formation. No significant difference in specific activity was observed between the WT and MUT recombinant enzymes present in the supernatant. The RmCBE WT and MUT recombinant enzymes were purified using single-step affinity chromatography, resulting in relatively pure proteins. Further characterization of the purified protein toward the SP-derived substrates was not possible due to time constraints. Future work could use both wild-type and mutant purified rRmCBE proteins to characterize their effects on the most frequently used synthetic pyrethroid acaricides, thereby elucidating the effect of the Asp374Asn mutation and whether it is indeed responsible for the observed resistant phenotype.

## Ethics and consent

Ethical approval and consent were not required

## Materials & correspondence

All correspondence and requests can be sent to ML.

## Data Availability

The CVSA *R. microplus* CBE encoding gene sequence is available at: GenBank. Accession OR124739. https://www.ncbi.nlm.nih.gov/nuccore/OR124739.1/ (Labuschagne, M., 2023).
