# Supplementary material for: Cloning, recombinant expression, and characterization of a Rhipicephalus microplus carboxylesterase
Source: Gates Open Res. 2024 Aug 19;8:87. [Version 1] doi: 10.12688/gatesopenres.16245.1 (PMC11813172; doi:10.12688/gatesopenres.16245.1)
Supplement: Peer review history

# Reviewer report: Reviewer response for version 1

**DOI:** 10.21956/gatesopenres.17609.r39103

**Published:** 2025-01-09

**Authors:** Anam Hammid

**Affiliations:** 1 University of Eastern Finland,, Kuopio, Finland

This manuscript presents the cloning, expression, and partial characterization of both the wild-type and a mutant (Asp374Asn) Rhipicephalus microplus carboxylesterase (RmCBE). The study investigates this enzyme because the Asp374Asn mutation in RmCBE might be linked to increased resistance to synthetic pyrethroid (SP) acaricides, which are crucial for controlling tick populations in the cattle industry.

 The researchers cloned the RmCBE gene from a susceptible R. microplus isolate and introduced the Asp374Asn mutation using site-directed mutagenesis. They successfully expressed both the wild-type and mutant RmCBE proteins in *Pichia pastoris* and purified them through immobilized metal affinity chromatography. The enzymatic activity of the recombinant proteins was then assessed using p-nitrophenyl acetate as a substrate. Both the wild-type and mutant enzymes could hydrolyze this substrate, with no significant difference in their specific activities.

 The authors suggest that the lack of difference in specific activity between the wild-type and mutant enzymes may be due to the small size of the p-nitrophenyl acetate substrate. They recommend future research to focus on characterizing the enzymes' activity towards SP-derived substrates, which are more likely to be affected by the Asp374Asn mutation.

 Overall, this manuscript is a well-executed study. The authors provide evidence that the Asp374Asn mutation does not affect the ability of RmCBE to hydrolyze p-nitrophenyl acetate. However, they cannot demonstrate that the mutation makes it a better or more active enzyme. Further studies are needed to determine whether this mutation affects the enzyme's ability to hydrolyze SPs.

 Suggestions:

 1. It is suggested to improve the conclusion and discussion section to highlight the significance of the study. This conclusion is not enough to justify the study. The more detailed conclusion and future work required in the subject should be highlighted.

 2. Please clarify Figure 7 and add more details on protein models. What is the source to design the structure? 

 3. In addition to enzymatic activity studies with p-nitrophenyl acetate substrate, additional inhibition studies would have been included in the study to validate the specific activity of the enzymes.

Is the work clearly and accurately presented and does it cite the current literature?

Yes

If applicable, is the statistical analysis and its interpretation appropriate?

Not applicable

Are all the source data underlying the results available to ensure full reproducibility?

Yes

Is the study design appropriate and is the work technically sound?

Yes

Are the conclusions drawn adequately supported by the results?

Yes

Are sufficient details of methods and analysis provided to allow replication by others?

Yes

Reviewer Expertise:

Molecular Biology, Protein chemistry, Proteomics

I confirm that I have read this submission and believe that I have an appropriate level of expertise to confirm that it is of an acceptable scientific standard.

# Reviewer report: Reviewer response for version 1

**DOI:** 10.21956/gatesopenres.17609.r39102

**Published:** 2025-01-07

**Authors:** Nadia A. Soliman

**Affiliations:** 1 Universities and Research Institutes Zone, Alexandria, Egypt

**Reviewer Report**

 I have reviewed the manuscript entitled "Cloning, Recombinant Expression, and Characterization of a *Rhipicephalus microplus* Carboxylesterase" (Code: F1KR00CDE Gates-VER17609-R).

**Summary of the Work:** This study investigates the *Rhipicephalus microplus* carboxylesterase (CBE), a protein involved in the hydrolysis of synthetic pyrethroids (SPs), which are linked to increased resistance to SP-based acaricides due to a non-synonymous mutation (Asp374Asn). The authors cloned the CBE gene and functionally expressed it in *Pichia pastoris* following the removal of the native signal peptide. Site-directed mutagenesis introduced the Asp374Asn substitution. Both wild-type and mutant CBEs were successfully expressed, characterized, and purified using affinity chromatography under native conditions. The findings offer valuable insights into the production of recombinant tick-derived CBE proteins and will aid further research into SP resistance in ticks.

**General Comments:** The manuscript presents valuable data; however, several areas could be improved to enhance clarity and readability. Specifically, the authors should consider revising and restructuring certain sentences to more clearly outline the sequence of experimental steps and avoid redundancy. This would improve the overall flow and comprehension of the text.

**Specific Comments:**

1. **Clarity and Structure:** The authors are encouraged to revise complex sentences to improve the logical flow and readability of the manuscript. This will help readers follow the experimental process more easily and reduce unnecessary repetition.
2. **Imidazole Interference:** The issue of imidazole interference in activity measurements is not sufficiently addressed. Imidazole in the elution buffer can affect substrate hydrolysis and skew the results. I recommend that the authors discuss strategies for removing imidazole from the samples before activity measurements. One potential solution could be the use of a gel filtration column, such as PD-10, or performing dialysis to remove imidazole from the protein samples. These methods would help mitigate the interference and enhance the accuracy of the activity assays.

Is the work clearly and accurately presented and does it cite the current literature?

Yes

If applicable, is the statistical analysis and its interpretation appropriate?

Not applicable

Are all the source data underlying the results available to ensure full reproducibility?

Yes

Is the study design appropriate and is the work technically sound?

Yes

Are the conclusions drawn adequately supported by the results?

Partly

Are sufficient details of methods and analysis provided to allow replication by others?

Yes

Reviewer Expertise:

Microbiologist and molecular biology biotechnology

I confirm that I have read this submission and believe that I have an appropriate level of expertise to confirm that it is of an acceptable scientific standard.

# Reviewer report: Reviewer response for version 1

**DOI:** 10.21956/gatesopenres.17609.r39096

**Published:** 2024-12-26

**Authors:** Shengwei Sun

**Affiliations:** 1 KTH Royal Institute of Technology, Stockholm, Sweden

In the present study, the author solved the cloning, expression, and characterization of an animal carboxylesterase. Both wide-type and the mutant Asp374Asn were cloned, expressed and purified using column method. The manuscript looks interesting, but a lot of issues need to be addressed. Therefore, I would not recommend the indexing of the current manuscript. Specific comments are shown below.

 1. In the method section, the author used a lot of molecular biology methods to do the cloning. In fact, this can be done by genetic synthesis in a efficient way. And a diagram of the cloning procedure is good for readers to understand the process. Moreover, some key technical parameters should be described in the method part. For example, how to purify the proteins is unclear. 

 2. For protein expression and purification, it is good to test the effect of different signal peptide on the protein expression extracellularly. The codon optimization is needed to be done before the cloning. 

 3. The p-nitrophenyl acetate is generally used for the characterization of the esterase activity. It does not make sense to use it to understand the resistance of SP-based acaricides in this study. The substrate should be a specific synthetic pyrethroid used for determining the differences between the wide-type CBE and the mutant Asp374Asn.  

 4. No statistical analysis was done in the study. This makes the data unreliable. 

 5. More discussion and published papers should be provided in the main text. Then it looks like a SCI paper.

Is the work clearly and accurately presented and does it cite the current literature?

No

If applicable, is the statistical analysis and its interpretation appropriate?

No

Are all the source data underlying the results available to ensure full reproducibility?

Partly

Is the study design appropriate and is the work technically sound?

No

Are the conclusions drawn adequately supported by the results?

No

Are sufficient details of methods and analysis provided to allow replication by others?

Partly

Reviewer Expertise:

Enzyme, protein engineering, applied microbiology, environmental microbiology

I confirm that I have read this submission and believe that I have an appropriate level of expertise to state that I do not consider it to be of an acceptable scientific standard, for reasons outlined above.

# Response

**Published:** 2024-12-27

**Authors:** Michel Labuschagne

**Affiliations:** Research Innovation, Clinglobal, Bloemfontein, Free State, South Africa

Response to reviewer:

 The work presented in this report was conducted as part of a larger study and the aim of this report was to express and secrete active *R. microplus* CBE using recombinant methods. The CBE was of interest due to historic data indicating that the Asp374Asn mutation was most likely responsible for the synthetic pyrethroid resistant phenotype observed. Due to time constraints we decided to publish this report on the cloning and functional expression of the *R. microplus* CBE genes (WT and MUT) in Gates Open Research to contribute towards the data available to the tick research community. This report will allow other researchers, interested in continuing to study the impact of the mutation, to recombinantly express and secrete active WT and MUT versions of the *R. microplus* CBE for further characterization. 

 Addressing the specific comments by the reviewer:

 1. The aim of the study was the functional expression of RmCBE. Not to perform high level expression of RmCBE for industrial application. Even though gene synthesis using codon optimization is an easy and efficient way to obtain the target gene in many geographies, we do not have access to such a local service and importation of synthetic constructs has a time-value that could not be afforded due to the time constrains regarding the project. The codon adaptation index (CAI) of the *R. microplus* sequence was 0.59, supporting the cloning methodology used in this work and allowed the amplification and fusion of the 3 exons in a single working day. The methodology is clearly described in the text.

 The reviewer also indicates that technical parameters, eg. protein purification is unclear. The methods section indicate clearly that we used 7.5 ml supernatant with using 1 ml ThermoScientific™ HisPur™ Ni-NTA Resin slurry and the batch-wise method as per the manufacturer’s recommendations under native

 conditions (Thermo Fisher Scientific; Catalogue number: 88221). The batch-wise method provided by the manufacturer clearly describe each step and the wash and elution volumes are expressed as resin bed volumes.

 2. Comment 2 refers to signal peptides and some codon optimization. Again, the aim was to perform functional secretion of recombinant CBE protein. Evaluation of several different signal peptides (other than the highly efficient alpha-factor) coupled with codon optimization should be used if you want to increase protein production yields if this was the aim of the study and this could be combined with high density cultivation of the recombinant protein production host using a bioreactor.

 3. The use of p-nitrophenyl acetate as substrate was so indicate the presence of esterase activity and confirm that the secreted protein resulted in a increased hydrolytic activity towards p-nitrophenyl acetate. The paper clearly states in the conclusion that "Further characterization of the purified protein toward the SP-derived substrates was not possible due to time constraints. Future work could use both

 wild-type and mutant purified rRmCBE proteins to characterize their effects on the most frequently used synthetic pyrethroid acaricides, thereby elucidating the effect of the Asp374Asn mutation and whether it is indeed responsible for the observed resistant phenotype" Performing SP-based activity assays was originally planned, but sourcing the specific analytical columns and substrates was not permitted due to time constrains - please keep in mind that all of the specialty components (standards and columns) are not readily available within South Africa. 

 4. The reviewer indicated that no statistical analysis was performed. The methods section indicate clearly that a one-way ANOVA was performed on the absorbance values, followed by a post hoc Tukey HSD test. We reported significance in the results section.

 5. The reviewer indicates more discussion of published papers should be provided in the main text. We included and discussed all published data on the *R. microplus* CBE.

# Reviewer report: Reviewer response for version 1

**DOI:** 10.21956/gatesopenres.17609.r38136

**Published:** 2024-10-10

**Authors:** Enrique Reynaud

**Affiliations:** 1 Universidad Nacional Autónoma de México, Cuernavaca, Mexico

This manuscript describes the cloning, expression, and partial characterization of the wild-type and a mutant (Asp374Asn) *Rhipicephalus microplus* carboxylesterase (RmCBE). The rationale for studying this enzyme, is that the Asp374Asn mutation in RmCBE may be associated with increased resistance to synthetic pyrethroid (SP) acaricides. This is a significant finding because SPs are a major class of acaricides, and resistance to these chemicals is a serious threat to the cattle industry.

 The authors cloned the RmCBE gene from a susceptible R. microplus isolate. They then used site-directed mutagenesis to introduce the Asp374Asn mutation into the gene. They successfully expressed both the wild-type and mutant RmCBE proteins in Pichia pastoris and purified the proteins using inmobilized metal affinity chromatography. Then the enzymatic activity of the recombinant proteins was characterized using p-nitrophenyl acetate as a substrate. They found that both the wild-type and mutant enzymes were able to hydrolyze this substrate, but there was no significant difference in their specific activities.

 The authors propose that the lack of difference in specific activity between the wild-type and mutant enzymes may be due to the small size of the p-nitrophenyl acetate substrate. They also  suggest that future work should focus on characterizing the activity of the enzymes toward SP-derived substrates, which are more likely to be affected by the Asp374Asn mutation.

 Overall, this manuscript is a well-executed study. The authors provide evidence that the Asp374Asn mutation does not affect the ability of RmCBE to hydrolyze p-nitrophenyl acetate, however they cannot demonstrate that the mutation makes it a better or more active enzyme. Further studies are needed to determine whether this mutation affects the enzyme's ability to hydrolyze SPs.

 Minor suggestions:

 The authors should include a more detailed discussion of the limitations of their study, such as the effect of the size of the substrate on the enzyme activity.

 The authors should consider including a figure showing the alignment of the CVSA RmCBE protein sequence with other RmCBE sequences from GenBank. This would help to illustrate the high degree of conservation among these proteins.

 While a 3D model is presented, the authors do not comment on what (if any) existing structural data was used to create their model (e.g. AlphaFold). Since the authors propose that the Asp374Asn mutation, located in the cap structure of the enzyme, might alter substrate binding pockets specific to SP-derived substrates, providing details on the model generation would strengthen the study.

Is the work clearly and accurately presented and does it cite the current literature?

Yes

If applicable, is the statistical analysis and its interpretation appropriate?

Not applicable

Are all the source data underlying the results available to ensure full reproducibility?

Yes

Is the study design appropriate and is the work technically sound?

Yes

Are the conclusions drawn adequately supported by the results?

Yes

Are sufficient details of methods and analysis provided to allow replication by others?

Yes

Reviewer Expertise:

Arthropod neurobiology, developmental neurobiology, acarology, physiology, molecuolar biology

I confirm that I have read this submission and believe that I have an appropriate level of expertise to confirm that it is of an acceptable scientific standard.
